# A 7.4-Bit ENOB 600 MS/s FPGA-Based Online Calibrated Slope ADC without External Components

**DOI:** 10.3390/s22155852

**Published:** 2022-08-05

**Authors:** Mengdi Zhang, Ye Zhao, Yong Chen, Paolo Crovetti, Yanji Wang, Xinshun Ning, Shushan Qiao

**Affiliations:** 1 Institute of Microelectronics of Chinese Academy of Sciences, Beijing 100029, China; zhangmengdi@ime.ac.cn (M.Z.); wangyanji20@mails.ucas.ac.cn (Y.W.); ningxinshun@ime.ac.cn (X.N.); qiaoshushan@ime.ac.cn (S.Q.); 2 University of Chinese Academy of Sciences, Beijing 100049, China; 3 State-Key Laboratory of Analog and Mixed-Signal VLSI and IME/ECE-FST, University of Macau, Macao 999078, China; ychen@um.edu.mo; 4 Department of Electronics and Telecommunications (DET), Politecnico di Torino, 10129 Torino, Italy; paolo.crovetti@polito.it

**Keywords:** FPGA, analog-to-digital converter (ADC), time-to-digital converter (TDC), effective number of bits (ENOB), differential nonlinearity (DNL), integral nonlinearity (INL)

## Abstract

A slope analog-to-digital converter (ADC) amenable to be fully implemented on a digital field programmable gate array (FPGA) without requiring any external active or passive components is proposed in this paper. The amplitude information, encoded in the transition times of a standard LVDS differential input—driven by the analog input and by the reference slope generated by an FPGA output buffer—is retrieved by an FPGA time-to-digital converter. Along with the ADC, a new online calibration algorithm is developed to mitigate the influence of process, voltage, and temperature variations on its performance. Measurements on an ADC prototype reveal an analog input range from 0.3 V to 1.5 V, a least significant bit (LSB) of 2.6 mV, and an effective number of bits (ENOB) of 7.4-bit at 600 MS/s. The differential nonlinearity (DNL) is in the range between −0.78 and 0.70 LSB, and the integral nonlinearity (INL) is in the range from −0.72 to 0.78 LSB.

## 1. Introduction

An analog-to-digital converter (ADC) is indispensable in many implementations, such as COMS sensor imaging [1], liquid helium environment [2], positron emission tomography (PET) [3], and so on. More particularly, analog-to-digital converters (ADCs) and time-to-digital converters (TDCs) are usually used in PET equipment to estimate the energy and arrival time of electrical signals from silicon photomultiplier tube (SiPM) detectors. Then, the computer tomography image reconstruction algorithm is used to generate the image reflecting the biological distribution in the body [4,5,6,7,8].

At present, precision TDCs implemented on a field programmable gate array (FPGA), with a resolution of fewer than 10 ps, are employed to acquire the time of arrival [9,10,11], whereas ADCs [12,13,14,15,16] in application-specific integrated circuits (ASICs) are used for energy measurements and are connected at PCB level to the FPGA, which performs the energy integration algorithm. Owing to a large number of SiPM detector channels, many external ADC chips are required, resulting in relevant system cost and power consumption, which seriously limit the system integration. Aiming to address this issue, slope ADCs based on TDC, which are amenable to being implemented on FPGA, have been proposed to measure the time of arrival and energy on the same FPGA chip.

In 2007 [17], Wu proposed an FPGA-based ADC operating at 22.5 MS/s with an ENOB of 6 bits. In that work, an LVDS comparator is used to detect the crossings of the input signal with the reference slope signal generated using external components, and a TDC is used to measure their timing. Homulle utilized parasitic capacitance of the pad and one external resistor to create the reference slope. The ADC achieved 200 MS/s and an ENOB of 6 by two tapped delay line TDCs [18]. To achieve a higher speed and higher ENOB, Homulle used a multiphase clock interpolation technique to sample the analog signal multiple times within a clock period. A 1.2 GS/s ADC (actually, three-time-interleaved 400 MS/s ADCs) with an ENOB of 6 bits was demonstrated, whereby seven external resistors were used [19]. However, the above studies also required external modules. The 600 MS/s 7-bit ENOB ADC without any external components is implemented in the ZYNQ Ultrascale+ [20]. This paper uses the output buffer (OBUF) and differential input buffer (DIFFINBUF) to replace the capacitor, resistor and analog comparator. One input of DIFFINBUF is connected to OBUF and the other is connected to the analog input. A digital output is generated when the amplitude of the analog input voltage exceeds the slope voltage. However, in order to maintain the precision and stability, the non-linear relationship between the amplitude of the analog input signal and time needs to be calibrated regularly.

In this work, a new slope ADC on FPGA based on TDC with online calibration is proposed. The proposed ADC generates the reference slope without requiring external components, taking advantage of the output resistance of a digital output buffer and its parasitic capacitance. Moreover, a 7.4-bit ENOB is achieved thanks to a new calibration strategy. The rest of the paper is organized as follows: the ADC architecture is introduced in Section 2; the proposed calibration strategy is then discussed in Section 3; while the experimental characterization of the proposed ADC, implemented on an Ultrascale+ FPGA is presented and compared with the state-of-the-art in Section 4. Some conclusions are finally drawn in Section 5.

## 2. System Architecture

**Figure 1:**
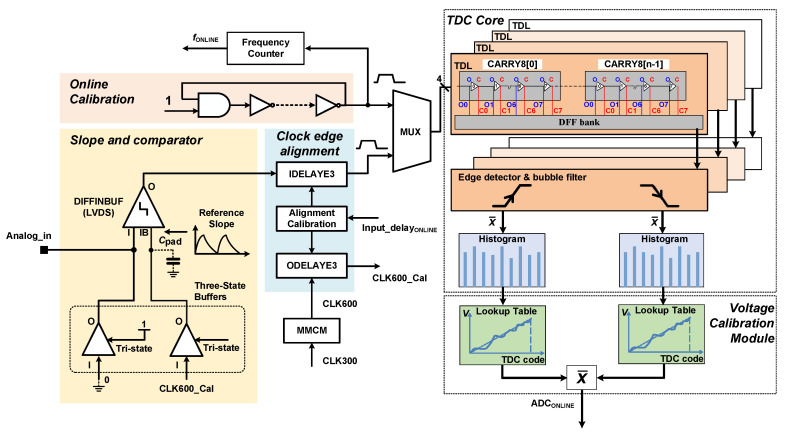
Overall architecture of the proposed FPGA-based ADC.

The architecture of the proposed ADC is shown in Figure 1. It consists of a comparator, a mixed-mode clock manager (MMCM), which generates a 600 MHz sampling clock, a TDC core consisting of four tapped delay line (TDL) TDCs, four edge detector and bubble filter modules, a bin-by-bin calibration module, a voltage calibration module, a clock edge alignment calibration module, and a new online calibration module. 

**Figure 2:**
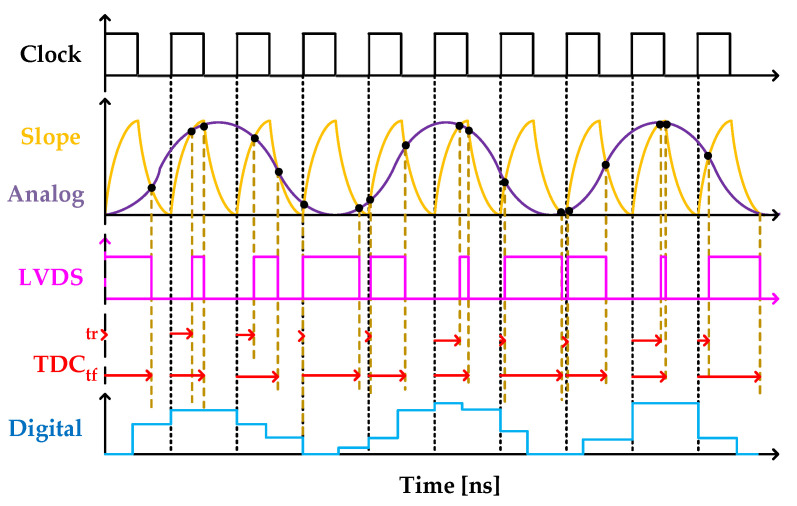
Timing diagram of our FPGA-based ADC. “Reproduced from [20]”.

The comparator module includes two single-ended output buffers OBUFT, and an LVDS comparator DIFFINBUF. The output resistance of the OBUFT with parasitic capacitance creates the slope signal for the ADC: one OBUFT is set to tri-state and its input terminal is driven by the 600 MHz system clock, and its output is connected to the N terminal of the analog signal input. The tri-state (T) terminal of the other OBUFT is connected to ‘1’, the input terminal is connected to ‘0’, and the output is connected to the O terminal of the analog signal. The ADC detects the positions in time of the rising and falling edges ( $t_{r}\;\mathrm{and}\;t_{f}$ ) of the LVDS comparator output via a TDC, as detailed in Figure 2.

In general, the parasitic capacitance on the pad Cpad is 5 pf. Therefore, the RC constant is 300 ps, which is less than half a clock (833 ps). Assuming that active $V_{u}$ charges capacitor C through resistance R , $V_{0}$ is the initial voltage value of the capacitor, $V_{u}$ is the voltage value after the capacitor is fully charged, and $V_{t}$ is the voltage value on the capacitor at time *t*, then the following calculation formula can be obtained:

(1) $$V_{t}=V_{0}+\left(V_{u}-V_{o}\right)\times \left[1-exp\left(-t/RC\right)\right]$$

If the initial voltage on the capacitor is 0, the formula is simplified as follows:

(2) $$V_{t}=\left(V_{u}-V_{o}\right)\times \left[1-exp\left(-t/RC\right)\right]$$

When t=RC , $V_{t}=0.63\;V_{u}$ ; t=2RC , $V_{t}=0.86\;V_{u}$ ; t=3RC , $V_{t}=0.95\;V_{u}$ . The $V_{u}$ is set to 1.8 V and the measured maximum value of ADC is 1.5 V ( $V_{t}=0.833\;V_{u}$ ). Therefore, the slope can be charged to 1.5 V within two time constants.

When the capacitor is fully charged, the capacitor C will discharge, that is, at time t, the voltage on the capacitor is as follows:

(3) $$V_{t}=V_{u}\times exp\left(-t/RC\right)$$

The two output buffers are set to have a 60 Ω output impedance and minimum (slowest) slew rate, thus output is slow enough to increase the time from 0 V to 1.8 V is about one-half of the clock period.

**Figure 3:**
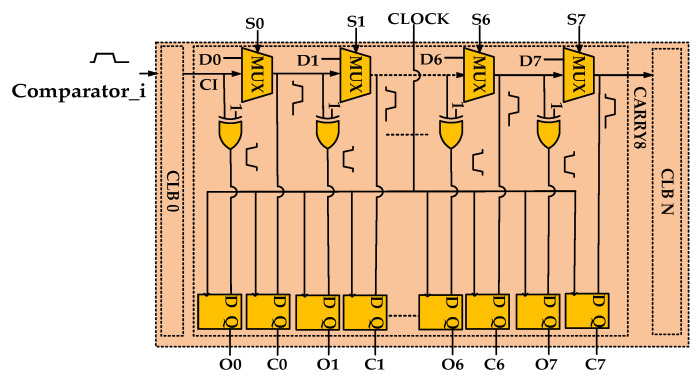
Detailed diagram of the CARRY8 block.

The TDC has been implemented by a tapped delay line featuring multiplexers (MUXs) as unit delay elements [21,22]. In the 16 nm UltraScale+ FPGAs used in this work, as Figure 3 shows, there are eight MUXs and eight XORs in the CARRY8 block [23,24]. The input CI of the first CARRY8 is connected to the output of the comparator and the CI of next level CARRY8 is connected to CO7 of a higher level. All DI configurations are 00000000 and SI configurations are 11111111. Once the coordinates of CARRY8 are constrained, all CARRY8 become a chain state arrangement, also known as TDL [25]. D Flip-Flop (DFF) samples C0–C7 (outputs of eight MUXs) and O0–O7 (outputs of eight XORs). To reduce routing uncertainty, CARRY8 primitive and DFF primitive are locked. Based on timing analysis, the delay of CARRY8 is 41 ps. In addition, considering the special coding circuit used, 60 CARRY8 elements are instantiated to obtain a delay greater than one clock cycle at 600 MHz. As the dedicated fast look-ahead carry logic architecture in the CARRY modules is prone to cause serious bubble problems and significant nonlinearity, the taps need to be reordered to improve the TDC linearity. Moreover, four chains are measured in parallel to improve the precision of TDC [26].

**Figure 4:**
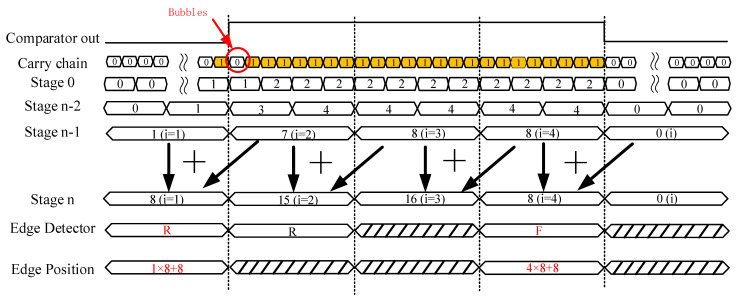
Timing diagram of the edge detector and bubble filtering. “Reproduced from [20]”.

The encoder module is used to convert the thermometer code into a binary code and to obtain the absolute position of the rising and falling edges of the LVDS comparator output, as illustrated in Figure 4. This work uses a special form to represent the edge detector and bubble filtering based on reference [20], instead of a generalized form. From stage 0 to stage *n* − 1, the thermometer code is converted into binary code through the pipeline full addition tree, which accepts the 960-bit binary array as an input and generates a 240-bit output. In the stage *n*, an overlapping sum S(n,i) is derived by the following:

(4) S(n,i)=S(n−1,i)+S(n−1,i+1)

where i is the *i*-th element of the stage *n*.

Once all sums are calculated, the rising edge and falling edge are detected by Algorithms 1. When the first rising edge and falling edge are determined, the number *i* and overlapping sum S(n,i) can also be obtained. The position Pos(i) of the rising edge and falling edge relative to the clock is obtained by the following formula:

(5) Pos(i)=i×8+S(n,i)

**Algorithms 1:** Edge Detection. “Reproduced from [20]”
**Step**

**Operation**
1
**if**

S(n,i+1)>8
 **then**
2  TranDir(i) 
←
 0     **// Falling edge**3 **if**

S(n,i−1)<8
 **then**4  TranValid 
←
 1     **// Valid Falling edge detection**5
**end if**
6 **else if** 
S(n,i+1)<8
 **then**7  TranDir(i) 
←
1     **// Rising edge**8 **if**

S(n,i−1)>8
 **then**
9  TranValid 
←
 1     **// Valid Rising edge detection**10  **end if**
11
**end if**
12
**end if**


## 3. Calibration Algorithm Flow

To improve the accuracy of the proposed ADC, four different calibration mechanisms, i.e., TDC bin-by-bin calibration, online calibration, ADC voltage calibration, and clock edge alignment calibration, are adopted in the proposed ADC and described in the following section.

### 3.1. TDC Bin-by-Bin Calibration

Affected by process, voltage, and temperature (PVT), the delay time of the digital circuit is not fixed. Therefore, it is necessary to calibrate the delay time of the carry chain before measurement. The delay time is usually a few picoseconds, making it difficult to calibrate the carry chain in the bit-by-bit scanning manner by generating a smaller time interval. There are two common calibration methods: one is the average calibration [27] and the other is the code density test calibration [28,29,30,31].

The average calibration method consists of measuring the signal with a known fixed time interval *T*. If the signal propagates *N* delay units in interval *T*, the average delay time τ is as follows:

(6) $$\tau =\frac{T}{N}$$

The average calibration method is applicable if the delay time consistency of the delay unit is good, and it is commonly used in ASIC-TDC. ASIC-TDC can make the delay time as consistent as possible through careful placement and routing. Owing to the influence of process, placement, and routing, the delay time between different delay cells varies greatly and has significant nonlinearity in FPGA devices. Using the average calibration method will bring large measurement errors. In addition, the tapped delay line designed in this paper is also affected by the carry look ahead. The code density test calibration is a bin-by-bin calibration method. Different from the average calibration method, the delay time of each delay cell can be calculated through the code density test. The principle of the code density test in TDC is similar to that in ADC [32]. The difference is that the code density test in ADC is the quantitative statistical analysis of voltage with random amplitude, while the code density test in TDC is the quantitative statistics with completely random time intervals.

**Figure 5:**
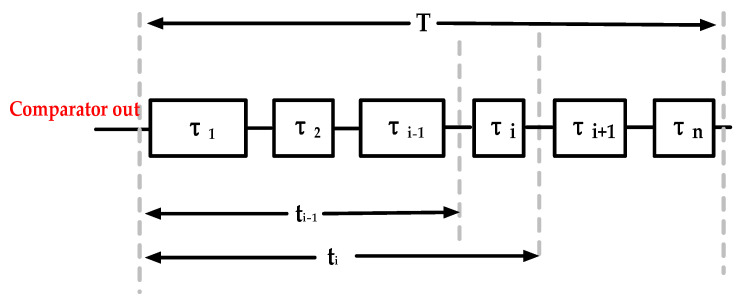
Block diagram of the code density test.

In this paper, the measurement range is a clock cycle; therefore, it is necessary to calibrate the delay cell used for inserting a clock cycle into the tapped delay line. The principle of the code density test in TDC is shown in Figure 5. If the comparator output is a random signal, the probability that the signal hits any phase point in the reference clock (0, *T*) is the same, that is, the time interval $t_{i}$ between the hit and the rising edge of the reference clock at any time is also completely random. The probability of random time interval is as follows:

(7) $$P(t<t_{i})=\frac{t_{i}}{T}$$

If the range of random time interval t is $\left(t_{i-1}<t<t_{i}\right)$ , the data of (i−1) tap in the delay line jump from 0 to 1 when the data are latched. As the time interval t is a random signal, it is called a random jump stopping at the (i−1) tap, and its probability is as follows:

(8) $$P(t_{i-1}<t<t_{i})=\frac{t_{i}}{T}\;$$

Suppose that, during *N* tests, the number of random jumps stopping at the (i−1) tap is h(i−1) , and its probability of occurrence is expressed as follows:

(9) $$P_{d}\left(i-1\right)=\frac{h_{i}}{N}$$

If the number of tests *N* is infinite, the following can be considered:

(10) $$P_{d}\left(i-1\right)=P(t_{i-1}<t<t_{i})$$

Based on (8) and (9),

(11) $$\tau_{i}=\frac{h\left(i-1\right)}{N}T$$

According to the statistical results, the delay time of each delay unit can be calculated in turn. When the time interval to be measured t ranges from $\left(t_{i-1}<t<t_{i}\right)$ , it can be approximately expressed as follows:

(12) $$t\approx t^{\prime }=\overset{i}{\underset{x=1}{\sum }}\tau_{x}+\epsilon =\frac{T}{N}\overset{i}{\underset{x=1}{\sum }}h\left(x-1\right)+\epsilon =\frac{T}{N}F\left(i-1\right)+\epsilon$$

where ϵ is quantitative valuation, F(i−1) is the cumulative probability distribution of the first *i* − 1 elements, and the standard deviation of measurement error is follows:

(13) $$\sigma^{2}=\frac{{\left(\tau_{i+1}-\epsilon \right)}^{3}+{\left(\epsilon \right)}^{3}}{3\tau_{i+1}}=\frac{{\left(\tau_{i+1}\right)}^{3}}{3}+{\left(\epsilon \right)}^{3}-\tau_{i+1}\epsilon$$

When $\epsilon =\frac{\tau_{i+1}}{2}$ , the standard variance was the smallest, and the minimum value is as follows:

(14) $$\sigma^{2}=\frac{{\left(\tau_{i+1}\right)}^{2}}{12}$$

In the actual code density test, the number of tests cannot be infinite. In *N* measurements, for any tap in the delay line, the random jump only stays at this tap and does not stay at this tap. Therefore, the statistical results of the random jump times of each tap h(i) obey the binomial distribution. The average value is as follows:

(15) $$\bar{h\left(i\right)}=Np_{d}\left(i\right)$$

The standard deviation is as follows:

(16) $$\sigma_{t}=\sqrt{Np_{d}\left(i\right)(1-p_{d}\left(i\right))}$$

As all samples are uncorrelated, that is, h(i) is uncorrelated, we can obtain the following from Formulas (11) and (12):

(17) $$\sigma_{t}=\frac{T}{N}\sqrt{\overset{i}{\underset{x=1}{\sum }}\sigma_{h\left(x-1\right)}^{2}+\frac{\sigma_{h\left(i\right)}^{2}}{2}}$$

Assuming there are *K* delay cells interpolated in a reference clock cycle, ideally, the delay times of these *K* delay units are the same, then $p_{d}\left(i\right)$ is equal, and $\sigma_{h\left(i\right)}^{2}$ is also equal:

(18) $$\sigma_{h}^{2}=\frac{N}{K}\left(1-\frac{1}{K}\right)$$

It can be seen from Formula (17) that $\sigma_{t}$ reaches the maximum value when *i = k*:

(19) $$\sigma_{t,max}=\frac{T}{N}\sqrt{\overset{k}{\underset{x=1}{\sum }}\sigma_{h}^{2}+\frac{\sigma_{h}^{2}}{2}}<\frac{T}{\sqrt{N}}\sqrt{1-\frac{1}{K}}$$

The system reference clock cycle *T* in this paper is 1.666 ns, and the maximum calibration error is less than 2 ps, so the calibration times need to meet *N* > 789,966. From the above analysis, it can be concluded that the calibration circuit needs to complete two functions; one is to generate the random signal, and the other is to statistically store the random jump times of each tap and establish the frequency distribution histogram.

**Figure 6:**
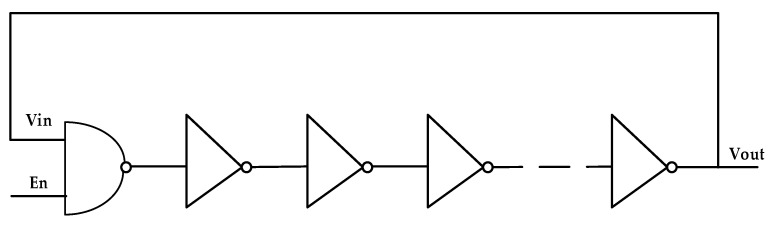
Block diagram of the inverter-based ring oscillator.

The hit is not related to the system reference clock, that is, they have different frequencies and no fixed-phase relationship. In digital circuits, the ring oscillator is usually used to generate a random signal. The ring oscillator is generally cascaded by an even number of inverters and one AND gate, and the output is fed back to the input to form a ring structure, as shown in Figure 6. Owing to the inherent delay of the gate circuit, it takes a certain time for the signal from Vin to Vout. When Vin and Vout are in an inverse-phase relationship with each other, the ring oscillator can vibrate, and the oscillation frequency is inversely proportional to the propagation delay from Vin to Vout.

**Figure 7:**
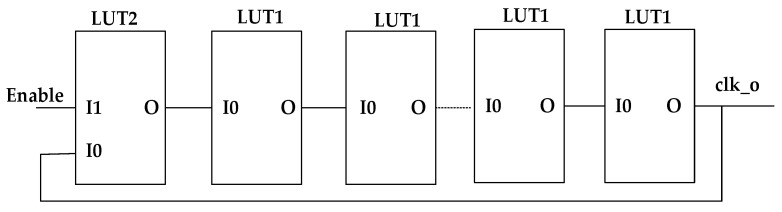
Schematic diagram of a ring oscillator constructed by a look-up table.

In Xilinx devices, there is no resistance-capacitance network and the delay time of the gate circuit is very short (generally tens of picoseconds). To generate low-frequency clock signals through ring oscillators, a large number of inverters are needed in FPGA. As the FPGA is based on look-up table technology, there are many lookup table resources inside it. Moreover, it is convenient to cascade them with each other. Therefore, this paper realizes the function of the inverter by configuring a lookup table to replace the inverter to form a ring oscillator. The ring oscillator composed of LUT1 and LUT2 is shown in Figure 7, including a total of one LUT2 and an even number of LUT1 (LUT1 and LUT2 are primitives in Xilinx FPGA). I0 is the one port of input, I1 is the anther port of input, and O is the port of output.

The input and output truth tables of LUT1 and LUT2 are shown in Table 1 and Table 2, respectively. It can be seen that, by configuring the internal storage value INT0–INT3 of LUT2 as “0010”, when I0 = “0”, LUT2 is not affected by the feedback signal, and the output is always “0”, so the ring oscillator stops oscillation. When I0 = “1”, the logic function of input I1 and output O of LUT2 is the same as that of an inverter. If I0 is “1”, O is “0”, and if I0 is “0”, O is “1”. By configuring the internal storage value INIT0–INIT 1 of LUT1 as “10”, LUT1 can realize the function of an inverter. When the input is “1”, the ring structure composed of LUT1 and LUT1 is equivalent to a ring oscillator composed of an odd number of inverters.

**Figure 8:**
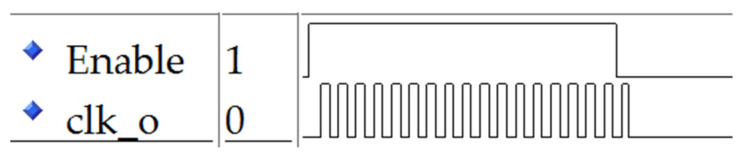
Timing simulation of the ring oscillator.

After synthesis, placement, and routing, the timing simulation results of the ring oscillator are shown in Figure 8. The ring oscillator vibrates when the enablement is 1 stops oscillating when the enablement is 0, and the output end is 0. By adjusting the number of LUT1, the oscillation frequency can be controlled.

To make the TDC more linear, the bin-by-bin calibration proposed by Wu [33] is adopted. For this purpose, the differential nonlinearity (DNL) and the integral nonlinearity (INL) are derived from the data histogram, and bin-by-bin calibration is performed based on the measured DNL and INL determined as follows:

(20) $$INL\left(k\right)=\overset{k-1}{\underset{i=1}{\sum }}DNL\left(i\right)+\frac{DNL\left(k\right)}{2}$$

**Figure 9:**
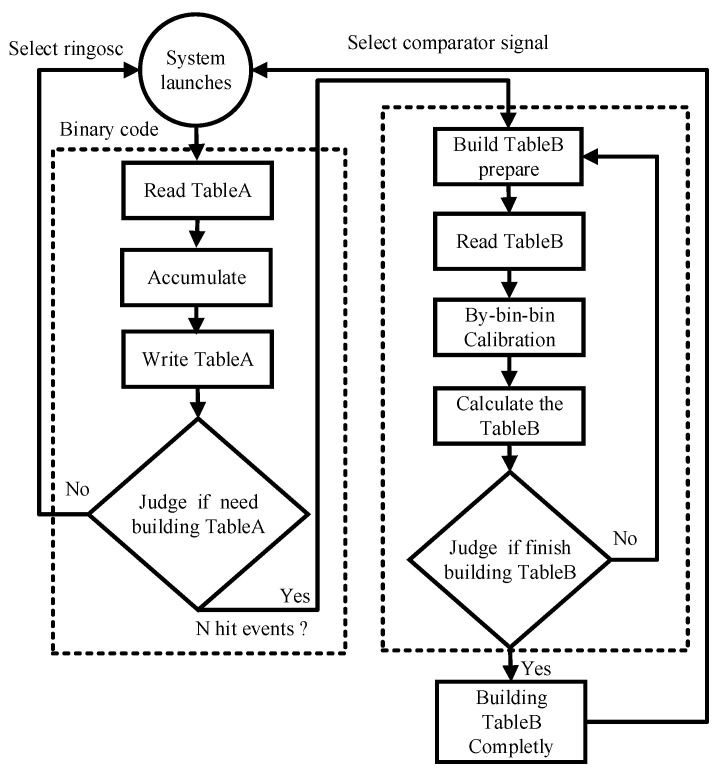
Flow chart of the bin-by-bin calibration.

Figure 9 shows the flow chart of the bin-by-bin calibration. Table A is port A and table B is port B of dual port SRAM. At system startup, the multiplexer selects the signal from the ring oscillator as the TDC input for initial calibration. First, *N* edge acquisitions are performed and the fine count associated with each binary code is retrieved and stored in Table A to perform a code density test, i.e., to estimate the width of each bin. In our design, *N* was chosen to be 1,024,000 as a good compromise between complexity and correction accuracy. After Table A is completely built, the following acquisitions are corrected with Table B for the bin-by-bin calibration, which takes clock cycles. For those 1,024,000 ring oscillator cycles and 1024 clock cycles, the bin-by-bin calibration is completed, then MUX selects the comparator signal and enters into the measurement mode, in which the value corresponding to each binary code is obtained from Table B.

### 3.2. Voltage Calibration

**Figure 10:**
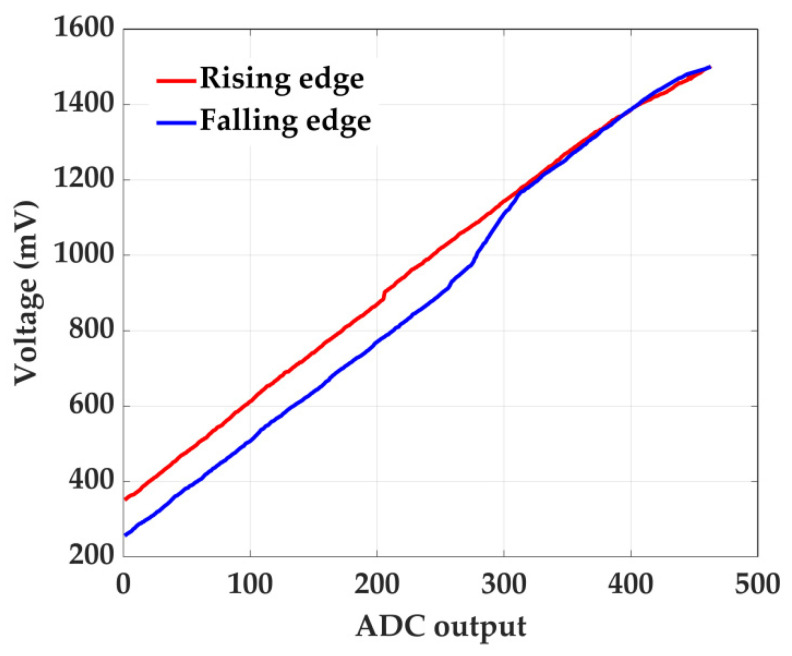
Voltage characteristic of rising and falling slope.

After the TDC calibration, a voltage calibration is performed to correct the non-linearity in the static characteristics of the ADC due to errors related to errors in the reference slope and in the comparator, and residual errors in the TDC. This is carried out using two lookup tables (one for the rising edge and one for the falling edge), implemented as BRAMs and initialized based on the acquisition of a triangular wave input. The content of such lookup tables for the ADC prototype considered in this paper is shown in Figure 10. In practice, such a test input can also be generated by a low-frequency FPGA-based DAC [34]. After 1,024,000 clock cycles, the measured falling and rising edges are fed into the LUTs, which provide the corrected value for the two edges at the next clock cycle.

### 3.3. Online Calibration

Besides the TDC and foreground ADC voltage calibration described thus far, online calibration is performed to track and compensate for voltage and temperature variations. In the formed FPGA devices, the doping concentration and width and length of the transistor can be regarded as fixed. At the same time, the low-voltage differential regulator (LDO) can provide low-noise power for the FPGA core, and the influence of power fluctuation can be ignored. Therefore, the amplitude change of the working voltage is generally small in the actual circuit, and the impact on the delay time is small. However, it is difficult to reduce the impact of temperature change on the FPGA in many applications. When the temperature changes, some parameters of the transistor (such as the leakage current and carrier migration speed) will change. The threshold voltage of the transistor decreases linearly with the temperature [35]. In different working environments, the temperature may vary by tens of degrees Celsius, so the main factor affecting the delay change of the delay chain is the ambient temperature.

In [29], it is considered that the delay time of all delay chains is basically affected by temperature changes. A special delay chain is used to monitor the influence of temperature on the delay time. Through many experiments, the corresponding relationship coefficient between temperature and code width is fitted to compensate for the influence of temperature on the delay time in real time, and XADC is used to observe the temperature of the FPGA chip in real time, Then, according to the real-time temperature of the chip, the corresponding coefficient is found to compensate for the influence of temperature on the time delay. Although this method can calibrate the influence of temperature on time delay in real time, it is too complex and the data in the look-up table are different every time the calibration is started, so the influence of temperature on time delay cannot be accurately compensated by looking up the corresponding coefficient between temperature and code width.

**Figure 11:**
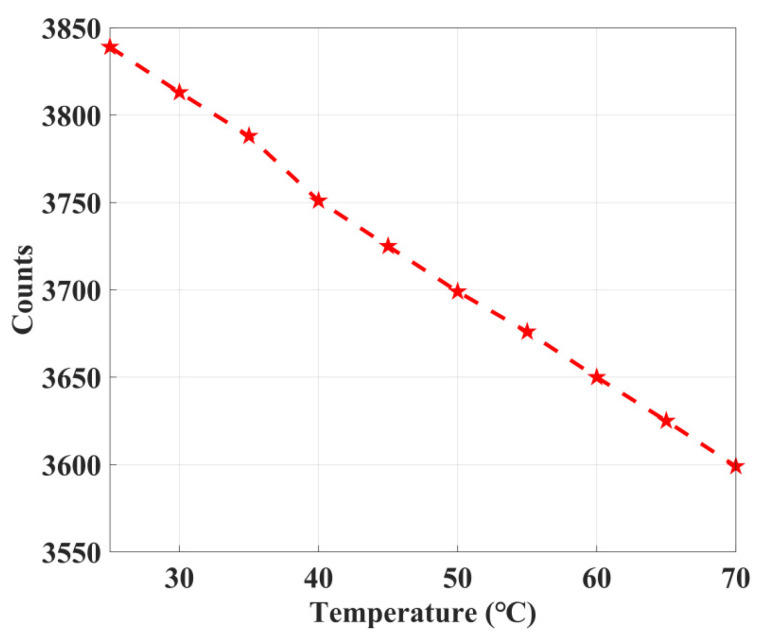
Dependence of ring oscillator frequencies on temperature.

For this purpose, an innovative approach, which consists of using a frequency counter to measure the ring oscillator frequency, is adopted. When the system is working, the frequency of the ring oscillator is measured and compared with that acquired at the beginning of the measurements. As the delay changes with voltage and temperature, the frequency of the ring oscillator also varies, thus making it possible to extrapolate its effect on the delay line of the TDC and compensate for it. We count the frequency of ring oscillator at a step length of 5 °C from 25 °C to 70 °C, as Figure 11 shows. The count decreases linearly with the increase of temperature.

In this paper, the ring oscillator is placed near each tapped delay line. The delay change of the delay cell is linearly updated by monitoring the frequency change of the ring oscillator to enable online temperature compensation. The principle of online temperature compensation is as follows: when the temperature changes, the frequency of the ring oscillator adjacent to the tapped delay line will also change, which is related to the change in delay time. Counting the frequency of the ring oscillator with a high-precision crystal oscillator can accurately calibrate the delay time and effectively reduce the delay error caused by temperature. The frequency count of the ring oscillator is approximately inversely proportional to the delay of the tapped delay line. The time delay $\tau_{i}^{\prime }$ after online calibration is expressed by (11):

(21) $$\tau_{i}^{\prime }=\frac{f_{\mathrm{off}}}{f_{\mathrm{on}}}\tau_{i}$$

where $f_{\mathrm{on}}$ is the frequency count measured during online calibration and $f_{\mathrm{off}}$ is the frequency count of the ring oscillator stored when starting calibration.

### 3.4. Clock Edge Alignment Calibration

To ensure the rising and falling edges appear in the same clock cycle, and to minimize the ADC offset, the reference pulse and the TDC sampling clock need to be aligned so that the pulse corresponds to the middle of the tapped delay line [9]. This condition is enforced in the proposed ADC by changing the input delay (IDELAYE3) or output delay (ODELAYE3) of the launching signal, as shown in Figure 1.

However, the delay of the device can be affected by PVT, especially when the clock frequency is very high. For robust operation, it is, therefore, necessary to change the value of the input or output delay automatically to track the changes in delay. In this design, this is accomplished with the same strategy adopted for online calibration.

## 4. Results

The proposed ADC is implemented in the ZCU104 demo board. The waveform generator Agilent N6705B was connected to the FPGA for static and dynamic ADC characterization.

Table 3 shows the full ADC used 21,980 LUTs, 34,105 registers, 627 CARRY8s, and 15 BRAMs.

**Figure 12:**
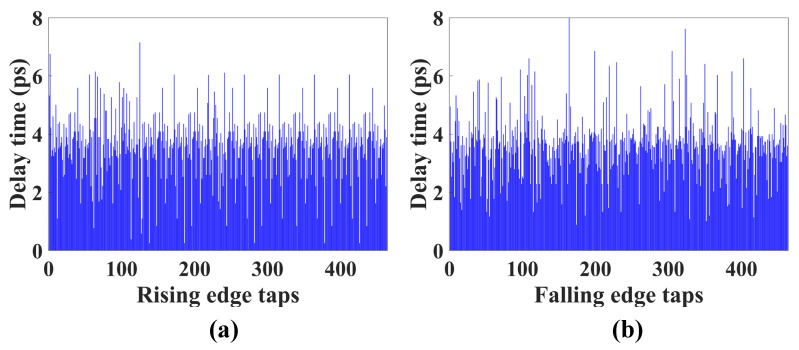
Measured histogram in the carry chain (**a**) rising edge; (**b**) falling edge.

Figure 12 shows the delay time of the carry chain. As the number of these carry elements is 463 for a 600 MHz clock, the average delay is 3.6 ps. Owing to the double sampling method, 463 × 2, i.e., 926 sampling points, are used for the ADC. The range and resolution of the ADC are related to the number of carrying elements and to the average delay of the TDC. As the dead band of the LVDS input is from 0 V to 0.3 V and 1.5 V to 1.8 V in the FPGA considered in this paper, the input voltage range of the ADC is 1.2 V. In the ADC prototype, the resolution of the ADC is 1.2 V/463 = 2.6 mV and its range *N* can be expressed in bits as follows:

(22) $$N=\log_{2}\frac{926*1.2V}{1.8V}=9.3$$

**Figure 13:**
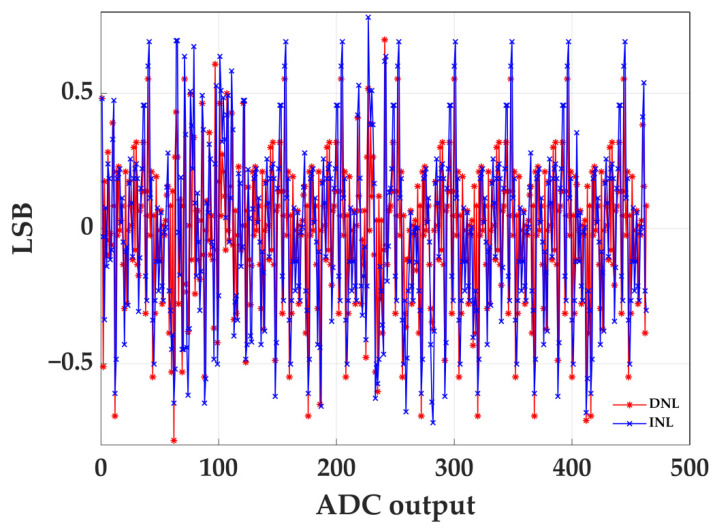
Measured DNL and INL of ADC.

Figure 13 shows the measured DNL and INL of the ADC; the DNL is within −0.78 and 0.70, while the INL is within −0.72 and 0.78. Although the DNL and INL of this design are not as good as in [16], this paper does not use any external devices, while seven external components are used in [16].

**Figure 14:**
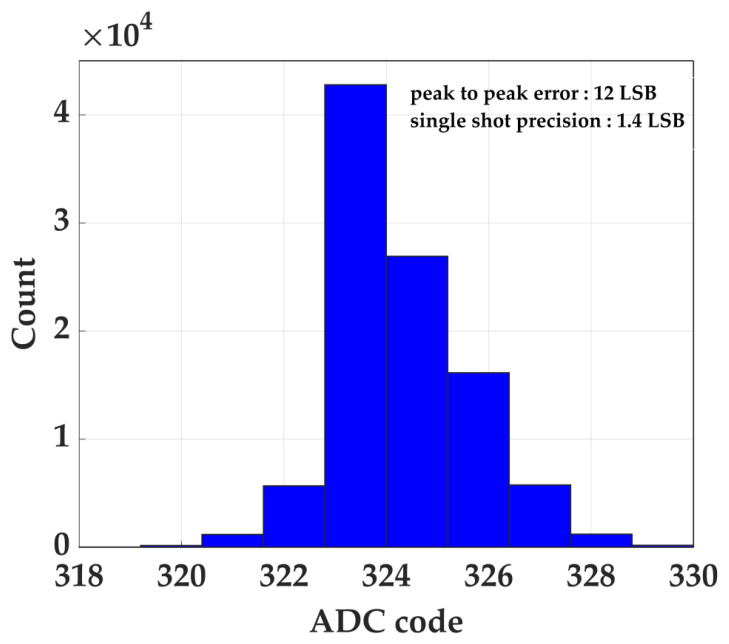
Single-shot measurement obtained by applying a DC voltage.

Figure 14 shows the histogram of single-shot ADC acquisitions under a 1.2 V DC input voltage; the peak-to-peak error is 12 LSB and the single-shot precision is 1.4 LSB. This shows that ADC has outstanding measurement precision.

**Figure 15:**
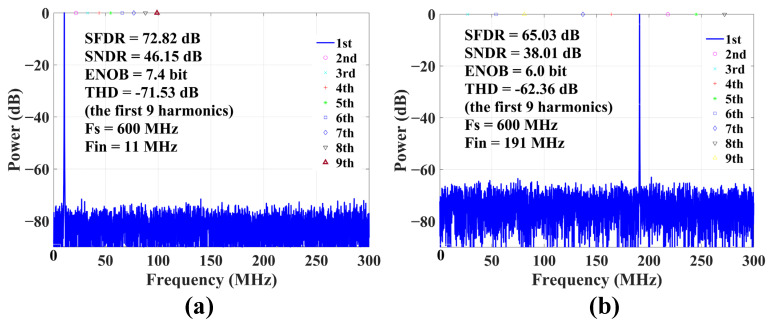
FFT of the 11 MHz (**a**) and 191 MHz; (**b**) 1 V_pk-pk_ sine wave for SNDR estimation.

Figure 15 shows the fast Fourier transform of the ADC output for the 1 V_pk-pk_ sine wave input at 11 MHz and 191 MHz, considered for signal-to-noise-and-distortion ratio (SNDR) and spurious-free dynamic range (SFDR) estimation. The SNDR (SFDR) is 46.15 dB (72.82 dB) at 11 MHz and 38.01 dB (65.03 dB) at 191 MHz.

**Figure 16:**
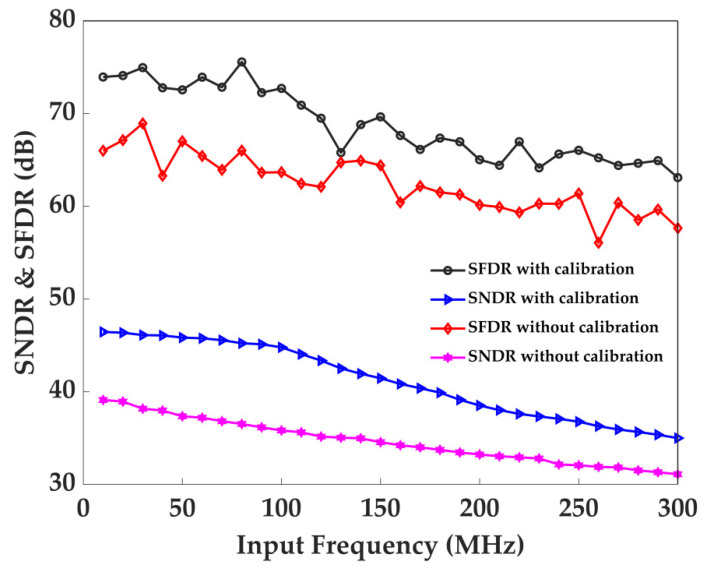
Measured SNDR and SFDR plots versus input frequency with or without online calibration.

The SNDR and SFDR are reported in Figure 16 versus the input signal frequency, revealing consistent performance up to 300 MHz (<10.85 dB SNDR degradation and <11.45 dB SFDR degradation at 300 MHz). It can be observed that the SNDR and SFDR of the ADC depend on the frequency of the input signal. This degradation can be traced back to the dynamic nonlinearity of the LVDS comparator.

**Figure 17:**
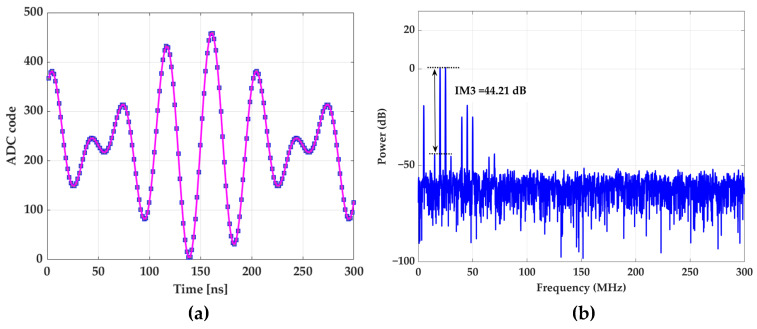
Two-tone test at 20 and 25 MHz. (**a**) Time domain; (**b**) frequency domain.

The two-tone intermodulation distortion is tested at 20 MHz and 25 MHz. The result is depicted in Figure 17 in the time and frequency domains. Besides significant power in third-order intermodulation distortion (IM3) components, more cross-coupled harmonics are seen.

**Figure 18:**
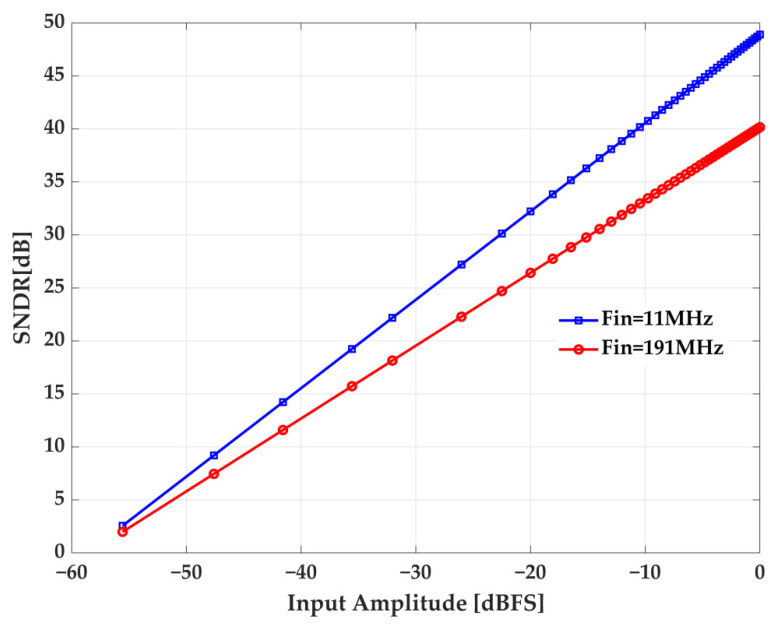
Measured SNDR versus input amplitude.

The ADC dynamic performance is analyzed by measuring SNDR versus input amplitude. The unit of abscissa dBFS is equal to $20log\left(\frac{A_{in}}{A_{full\_range}}\right)$ , where $A_{in}$ is the input amplitude and $A_{full\_range}$ is the full range. As Figure 18 shows, SNDR increases linearly when the input signal amplitude increases.

Table 4 benchmarks the ADC proposed in this work with other state-of-the-art FPGA-ADCs. The work of [19] operates at 400 MS/s with 6-bit ENOB. The work of [2] achieved an 800 MS/s sample rate at only 3.9-bit ENOB for a 100 MHz analog signal input. The work of [20] achieved a 600 MS/s sample rate at 7-bit ENOB for a 1 MHz analog signal input. The ADC presented in this work achieves the highest ENOB, while operating at a sampling rate higher than in [18,19,36]. Moreover, unlike the other FPGA ADCs in Table 1, the proposed ADC does not need external components.

## 5. Conclusions

This work presents an ADC implemented in FPGA without any external elements. The ADC creates the reference slope inside the FPGA and compares it to an analog signal with a comparator. The TDC is utilized to measure the absolute position of the rising and falling edge of this comparator output. Additional calibrations are implemented to increase the ENOB of the ADC. Based on measured results, the ADC has an LSB of 2.6 mV, showing a DNL within [−0.78, 0.70] and an INL within [−0.72, 0.78]. Thanks to the implemented calibration strategies, the proposed FPGA ADC operates at 600 MS/s with an ENOB of 7.4-bit, which is the highest reported for an FPGA-based ADC at a comparable sample rate.

## Figures and Tables

**Table 1:** LUT2 input/output truth table.

Input		Output	INIT
I1	I0	O	Internal storage
0	0	0	INIT (0) = 0
0	1	0	INIT (1) = 0
1	0	1	INIT (2) = 1
1	1	0	INIT (3) = 0

**Table 2:** LUT1 input/output truth table.

Input	Output	INIT
I	O	Internal storage
0	1	INIT (0) = 1
1	0	INIT (1) = 0

**Table 3:** Utilization of the ADC.

Element	Used	Available
LUT	21,980	230,400
Register	34,105	460,800
CARRY8	627	28,800
BRAM	15	312

**Table 4:** Performance summary and comparison of state-of-the-art FPGA-based ADCs.

	NSSC’07 Wu [17]	ACM’15 Homulle [18]	TCASI’16 Homulle [19]	HPEC’18 Xiang [2]	TRPMS’22 Ma [36]	ACM’21 Leuenberger [20]	This Work
Device	Altera cyclone	Spartan-6	Artix-7	Artix-7	Kintex-7	Ultrascale+	Ultrascale+
External components	4	3	7	1	1	0	0
Sample rate (MS/s)	22.5	200	400	800	25	600	600
Dynamic range (V)	0–3.3	0–2.5	0.9–1.6	0–3.0	0.11–1	0.15–1.45	0.3–1.5
LSB (mV)	52	17	3	N/A *	N/A *	2	2.6
Single-shot prec. (LSB)	N/A *	2	1.1	N/A *	N/A *	N/A *	1.4
ENOB	N/A *	6 bit (@1 MHz)	6 bit (@1 MHz)	3.9 bit (@100 MHz)	5.8 bit (@1 MHz)	7 bit (@1 MHz)	7.4 bit (@11 MHz)
DNL (LSB)	N/A *	[−0.9, 1.4]	[−0.75, 1.04]	[−0.5, 0.6]	N/A *	[−0.9, 0.9]	[−0.78, 0.70]
INL (LSB)	N/A *	[−1.1, 1.6]	[−0.36, 0.52]	[−0.2, 0.5]	N/A *	[−1.1, 0.9]	[−0.72, 0.78]
FOM (fJ/c-s) **	N/A *	32,031	29,297	37,926	N/A *	N/A *	10,831

* N/A means that this parameter is not mentioned in the reference. ** FOM = power/(Fs·2^ENOB^).

## Data Availability

The data presented in this study are available upon request from the corresponding author. The data are not publicly available for privacy reasons.
