# Correction: Zhang et al. A 7.4-Bit ENOB 600 MS/s FPGA-Based Online Calibrated Slope ADC without External Components. *Sensors* 2022, *22*, 5852

**DOI:** 10.3390/s22228936

**Published:** 2022-11-18

**Authors:** Mengdi Zhang, Ye Zhao, Yong Chen, Paolo Crovetti, Yanji Wang, Xinshun Ning, Shushan Qiao

**Affiliations:** 1 Institute of Microelectronics of Chinese Academy of Sciences, Beijing 100029, China; 2 University of Chinese Academy of Sciences, Beijing 100049, China; 3 State-Key Laboratory of Analog and Mixed-Signal VLSI and IME/ECE-FST, University of Macau, Macao 999078, China; 4 Department of Electronics and Telecommunications (DET), Politecnico di Torino, 10129 Torino, Italy

The authors wish to correct the following errors in the original paper [1].

## Text Correction

There was an error in the original publication (the wrong use of uniquely).

The following correction has been made to Section 1. Introduction, Paragraph 4:

The proposed ADC generates the reference slope without requiring external components, taking advantage of the output resistance of a digital output buffer and its parasitic capacitance.

## Missing Citation

In the original publication, Reference [20] was not cited. The citation has now been inserted. With this correction, the order of some references has been adjusted accordingly.

- In Section 1. Introduction, Paragraph 3

The 600 MS/s 7-bit ENOB ADC without any external components is implemented in the ZYNQ Ultrascale+ [20]. This paper uses the output buffer (OBUF) and differential input buffer (DIFFINBUF) to replace the capacitor, resistor and analog comparator. One input of DIFFINBUF is connected to OBUF and the other is connected to the analog input. A digital output is generated when the amplitude of the analog input voltage exceeds the slope voltage. However, in order to maintain the precision and stability, the non-linear relationship between the amplitude of the analog input signal and time needs to be calibrated regularly.

- In the caption of Figure 2

Figure 2. Timing diagram of our FPGA-based ADC. “Reproduced from [20]”.

- In the caption of Figure 4

Figure 4. Timing diagram of the edge detector and bubble filtering. “Reproduced from [20]”.

- In the caption of Algorithms 1

Algorithms 1: Edge Detection. “Reproduced from [20]”.

- In Section 2. System Architecture, Paragraph 9

This work uses a special form to represent the edge detector and bubble filtering based on Reference [20], instead of a generalized form.

- In Section 4. Results, Paragraph 10

The work of [20] achieved a 600 MS/s sample rate at 7-bit ENOB for a 1 MHz analog signal input.

- In the Acknowledgments

We thank Lucas Leuenberger, author of [20], whose help is invaluable in improving our thinking and work to a higher level.

- In the Reference Section, a new reference of [20] was added:

20. Leuenberger, L.; Amiet, D.; Wei, T.; Zbinden, P. An FPGA-based 7-ENOB 600 MSample/s ADC without any External Components. In Proceedings of the 2021 ACM/SIGDA International Symposium on Field-Programmable Gate Arrays, New York, NY, USA, 28 February–2 March 2021; pp. 240–250.

## Error in Figure/Table

In the original publication, there was a mistake in Table 4. Reference [20] is not used as a comparison. The corrected Table 4 appears below.

The authors state that the scientific conclusions are unaffected. This correction was approved by the Academic Editor. The original publication has also been updated.

## Figures and Tables

**Table 4:** Performance summary and comparison of state-of-the-art FPGA-based ADCs.

	NSSC’07 Wu [17]	ACM’15 Homulle [18]	TCASI’16 Homulle [19]	HPEC’18 Xiang [2]	TRPMS’22 Ma [36]	ACM’21 Leuenberger [20]	This Work
Device	Altera cyclone	Spartan-6	Artix-7	Artix-7	Kintex-7	Ultrascale+	Ultrascale+
External components	4	3	7	1	1	0	0
Sample rate (MS/s)	22.5	200	400	800	25	600	600
Dynamic range (V)	0–3.3	0–2.5	0.9–1.6	0–3.0	0.11–1	0.15–1.45	0.3–1.5
LSB (mV)	52	17	3	N/A *	N/A *	2	2.6
Single-shot prec. (LSB)	N/A *	2	1.1	N/A *	N/A *	N/A *	1.4
ENOB	N/A *	6 bit (@1 MHz)	6 bit (@1 MHz)	3.9 bit (@100 MHz)	5.8 bit (@1 MHz)	7 bit (@1 MHz)	7.4 bit (@11 MHz)
DNL (LSB)	N/A *	[−0.9, 1.4]	[−0.75, 1.04]	[−0.5, 0.6]	N/A *	[−0.9, 0.9]	[−0.78, 0.70]
INL (LSB)	N/A *	[−1.1, 1.6]	[−0.36, 0.52]	[−0.2, 0.5]	N/A *	[−1.1, 0.9]	[−0.72, 0.78]
FOM (fJ/c-s) **	N/A *	32,031	29,297	37,926	N/A *	N/A *	10,831

* N/A means that this parameter is not mentioned in the reference. ** FOM = power/(Fs·2^ENOB^).
